# Integrative Single‐Cell Transcriptomics and Network Pharmacology Analysis of Xiao Qing Long Tang in Pediatric Cough‐Variant Asthma

**DOI:** 10.1155/ijog/2536457

**Published:** 2026-04-03

**Authors:** Mingming Cui, Qianqian Li, Xue Ding, Mengjie Zhou, Ximeng Lou, Lihong Xia

**Affiliations:** ^1^ Department of Pediatrics, First Clinical College, Shandong University of Traditional Chinese Medicine, Jinan, 250000, Shandong, China, sdutcm.edu.cn; ^2^ Department of General Internal Medicine, Jinan Shizhong People’s Hospital, Jinan, 250002, Shandong, China; ^3^ Department of Pediatrics, Affiliated Hospital of Shandong University of Traditional Chinese Medicine, No. 16369 Jingshi Road, Jinan, 250000, Shandong, China, sdutcm.edu.cn

**Keywords:** airway epithelium, biomarkers, cellular heterogeneity, developmental trajectory, macrophage polarization, pediatric asthma, single-cell RNA sequencing, transcriptomics

## Abstract

**Background:**

Pediatric cough‐variant asthma is characterized by chronic airway inflammation and epithelial dysfunction driven by complex cellular interactions and molecular regulatory networks. Understanding the cellular heterogeneity and molecular mechanisms underlying pediatric asthma pathogenesis remains challenging. Single‐cell RNA sequencing (scRNA‐seq) provides unprecedented resolution to dissect the molecular landscape of asthmatic airways at the individual cell level.

**Methods:**

We performed a comprehensive single‐cell transcriptomic analysis of airway epithelial tissues from pediatric asthma patients using the 10x Genomics platform. Following stringent quality control, dimensionality reduction analysis, and cell clustering, we identified distinct cell populations and characterized their molecular signatures. Developmental trajectory analysis was performed using the Monocle algorithm, and functional enrichment analysis was conducted to elucidate biological pathways. Additionally, network pharmacology was employed to explore the multitarget mechanisms of the traditional Chinese medicine formula Xiao Qing Long Tang (XQLT) in treating asthma.

**Results:**

We successfully constructed a single‐cell atlas of pediatric asthma airway epithelium, identifying seven major cell subtypes: eosinophil cells, basal cells, ciliated cells, goblet cells, club cells, ionocyte cells, and deuterosomal cells. High‐resolution analysis revealed 23 distinct macrophage subpopulations (M1–M23) with unique transcriptional profiles. Key genes including KRT16, CXCL5, MMP10, ADAM12, and MALAT1 showed cell type–specific expression patterns. Pseudotime trajectory analysis revealed aberrant differentiation pathways from basal cells to specialized epithelial cells. Functional enrichment analysis highlighted inflammatory responses, immune system processes, and tissue remodeling as predominant biological processes in asthmatic airways. The network pharmacology analysis further identified 124 common targets, revealing that XQLT exerts multicomponent, synergistic therapeutic effects through core hubs such as NFE2L2 and NOS2.

**Conclusions:**

This comprehensive single‐cell transcriptomic atlas provides novel insights into the cellular heterogeneity and molecular mechanisms of pediatric asthma. The identification of cell type–specific gene expression patterns and developmental trajectories offers potential targets for precision therapeutic interventions.

## 1. Introduction

Asthma affects approximately 300 million people worldwide and represents one of the most common chronic diseases in children, with prevalence rates continuing to rise globally. Pediatric asthma is characterized by airway inflammation, bronchial hyperresponsiveness, and airway remodeling, leading to recurrent episodes of wheezing, breathlessness, chest tightness, and coughing [1–3]. Despite significant advances in understanding asthma pathophysiology, the heterogeneous nature of this disease and the complex interplay between genetic, environmental, and immunological factors continue to challenge our ability to develop personalized therapeutic strategies.

The airway epithelium serves as the first line of defense against inhaled pathogens, allergens, and environmental irritants, playing crucial roles in barrier function, mucociliary clearance, and immune regulation [4–6]. In asthmatic patients, the airway epithelium undergoes significant structural and functional alterations, including epithelial barrier dysfunction, enhanced mucus production, impaired ciliary function, and aberrant repair responses. These changes contribute to the characteristic features of asthma, including airway inflammation, bronchial hyperresponsiveness, and progressive airway remodeling.

Traditional bulk RNA sequencing approaches have provided valuable insights into the transcriptional changes associated with asthma, but they fail to capture the cellular heterogeneity and cell type–specific responses that characterize this complex disease. The emergence of single‐cell RNA sequencing (scRNA‐seq) technology has revolutionized our ability to dissect the molecular landscape of disease at unprecedented resolution, enabling the identification of rare cell populations, characterization of cellular states, and reconstruction of developmental trajectories [7–9].

Recent single‐cell studies in asthma have begun to unveil the remarkable cellular diversity within asthmatic airways, revealing distinct epithelial cell populations with specialized functions and unique molecular signatures. However, comprehensive single‐cell atlases of pediatric asthma airway epithelium remain limited, and the molecular mechanisms underlying disease‐specific cellular heterogeneity are not fully understood.

In this study, we present a comprehensive single‐cell transcriptomic analysis of airway epithelial tissues from pediatric asthma patients, aiming to (1) construct a detailed cellular atlas of the asthmatic airway epithelium, (2) characterize the molecular signatures of distinct cell populations, (3) elucidate developmental trajectories and cellular differentiation patterns, (4) identify disease‐relevant gene expression networks and signaling pathways, and (5) provide molecular insights for potential therapeutic targets.

## 2. Methods

### 2.1. Study Design and Data Sources

This study employed scRNA‐seq analysis to investigate the transcriptomic landscape of airway epithelial tissues in asthma. Publicly available scRNA‐seq datasets were obtained from the NCBI Gene Expression Omnibus (GEO), including GSE164015 and GSE193816, which comprise airway epithelial single‐cell transcriptomes derived from asthma patients under different experimental conditions. After data integration and quality control, the combined dataset included approximately 29 samples from 12 donors, yielding ∼75,000 cells for downstream analyses [10–12]. Cell Ranger software (Version 6.1.2) was used for processing raw sequencing data, including sequence alignment, gene counting, and cell barcode identification. Strict quality control was performed on single‐cell data, with assessment metrics including the number of genes detected per cell, total RNA counts, and mitochondrial gene expression ratios. Scatter plot analysis was used to examine the correlation between gene detection numbers and total RNA counts across cells, evaluating data quality and batch effects. Specifically, cells were filtered based on the number of detected genes per cell, total UMI counts, and the proportion of mitochondrial gene expression. Cells with very low gene counts (< 200 genes), excessively high transcript counts, or mitochondrial gene proportions exceeding approximately 15% were excluded. The relationship between the detected gene numbers and total RNA counts across cells was examined to assess overall data quality and technical consistency. Highly biologically variable genes were identified through the relationship between average expression levels and standardized variance. The average expression level and expression coefficient of variation for each gene across all cells were calculated, screening genes with both high expression and high variability as feature genes for subsequent analyses. These highly variable genes included epithelial cell differentiation‐related genes KRT16 (keratin 16), KRT17 (keratin 17), and KRT14 (keratin 14), as well as inflammation‐related genes SPRR3 (small proline‐rich protein 3) and MSMB (beta‐microseminoprotein), providing a molecular basis for understanding cellular heterogeneity in asthma pathological processes.

### 2.2. Dimensionality Reduction Analysis and Cell Clustering

Principal component analysis (PCA) was performed on the highly variable gene expression matrix for dimensionality reduction. The first 50 principal components were calculated, with the optimal number of principal components determined using the elbow method and variance contribution rates. The number of PCs retained for downstream analysis was determined based on a combination of elbow plot inspection and variance‐explained patterns, which indicated that the first approximately 15 PCs captured the majority of biologically relevant variation in the dataset. These selected PCs were subsequently used to construct a cell–cell shared nearest neighbor (SNN) graph, providing the basis for graph‐based clustering. Based on PCA results, a cell‐to‐cell adjacency graph was constructed, providing an optimized data structure for subsequent cell clustering and visualization analysis. Uniform Manifold Approximation and Projection (UMAP) was used for nonlinear dimensionality reduction visualization, projecting high‐dimensional gene expression data into two‐dimensional space. The Leiden algorithm was employed for graph clustering, with optimal clustering results obtained by adjusting resolution parameters. UMAP analysis successfully separated cell populations with different transcriptional characteristics into relatively independent distribution regions, establishing a foundation for cell type identification and functional annotation.

### 2.3. Cell Type Identification and Annotation

Cell type annotation was performed based on cluster‐specific marker genes identified by differential expression analysis. Marker genes were cross‐referenced with the published airway cell marker databases and relevant literature to ensure annotation accuracy. At the global level, cells were first classified into major airway microenvironment compartments, including epithelial‐related populations and immune or stromal cell types, consistent with the overall clustering structure. For finer characterization, epithelial‐associated clusters were subsequently extracted and reanalyzed at higher resolution, enabling the identification of epithelial subtypes such as basal cells, ciliated cells, goblet cells, club cells, ionocyte cells, and deuterosomal cells, as well as eosinophil‐enriched populations. The relative proportions of annotated cell types were calculated as a descriptive measure to support downstream analyses.

### 2.4. Developmental Trajectory Reconstruction and Dynamic Analysis

The Monocle algorithm was used to reconstruct cellular developmental trajectories, analyzing the differentiation process of airway epithelial cells. Using basal cells as the trajectory starting point, cell state transitions and differentiation pathways were traced. Pseudotime series were constructed to quantify the relative positions of cells along the developmental trajectory. Trajectory analysis revealed the continuous differentiation process from multipotent stem cells to specialized cells, which may be aberrant under asthmatic pathological conditions. Dynamic changes in gene expression along the developmental trajectory were analyzed, identifying gene modules that exhibited significant expression level changes during cell differentiation. Hierarchical clustering analysis was used to classify coexpressed genes into different expression modules, with each module representing specific biological functions or regulatory networks. These gene modules reflected key molecular events during cell differentiation, providing molecular mechanistic explanations for understanding asthma‐related cell state transitions.

### 2.5. In‐Depth Analysis of Macrophage Subpopulations

Macrophage‐associated cells were extracted from the global dataset and subjected to higher‐resolution clustering to characterize intrapopulation heterogeneity. Clustering resolution parameters were increased to enable finer stratification, resulting in the identification of 23 macrophage‐associated transcriptional clusters (M1–M23). Differential gene expression analysis was performed to identify marker genes for each macrophage cluster. To visualize cluster‐specific gene expression patterns, representative marker genes were summarized using violin plots. Macrophage clusters were further examined with reference to established macrophage polarization marker genes to describe activation‐related features. This analytical framework was used to characterize macrophage heterogeneity at the transcriptional level without presupposing fixed functional categories, supporting downstream interpretation of macrophage‐related programs in asthma.

### 2.6. Functional Enrichment Analysis and Pathway Resolution

Systematic GO functional enrichment analysis was performed on differentially expressed genes, encompassing three levels: biological processes, cellular components, and molecular functions. Enrichment significance was evaluated using the hypergeometric test, and multiple‐testing correction was applied using the Benjamini–Hochberg method, with an adjusted *p* value of < 0.05 considered statistically significant. Only GO terms containing at least five genes were retained for downstream interpretation to reduce the influence of sparsely annotated categories. The KEGG pathway database was used for signal pathway enrichment analysis, identifying classical signaling pathways closely related to asthma pathogenesis, and pathways with an adjusted *p* value of < 0.05 were considered significant [13–15]. Association networks between functional modules were constructed, analyzing interactions between different biological processes and molecular functions. Circular network diagrams were used to visualize complex functional associations, with connecting lines of different colors representing the strength of interfunctional relationships. This systematic pathway analysis provided a comprehensive molecular atlas for understanding asthma molecular mechanisms and searching for potential therapeutic targets.

### 2.7. Network Pharmacology Analysis and Target Identification

This study employed network pharmacology methods to systematically analyze the potential molecular mechanism of “Xiao Qing Long Tang” (XQLT) in treating asthma. First, active compounds and their predicted targets for each constituent herb of XQLT were retrieved from the BATMAN‐TCM database. Compound–target associations were screened according to BATMAN‐TCM–recommended criteria, with a target prediction score of ≥ 20 and an adjusted *p* value of < 0.05 retained for subsequent analysis. When pharmacokinetic information was available, basic ADME‐related properties, including oral bioavailability and drug‐likeness indices, were used as reference parameters to exclude compounds with low predicted bioavailability.

All predicted targets were standardized to official gene symbols using the UniProt database. At the same time, using multiple disease databases (GeneCards and OMIM), disease targets related to “asthma” were collected. For GeneCards, targets with a relevance score of ≥ 10 were retained to reduce nonspecific associations. Drug‐related targets and disease‐related targets were intersected to identify common targets, which were considered candidate targets for downstream network analysis. The overlap between drug and disease target sets was visualized using the VennDiagram package in R (Version 4.0).

Subsequently, construct an interaction network of “herb–compound–common target,” and visualize it using the Cytoscape software (Version 3.8.0). The nodes in the network represent herbs, compounds, or targets, and the edges represent the interaction relationships between them. Perform topological analysis on the network and calculate the degree of nodes to identify the key hub targets.

To further analyze the contributions of each component, we conducted multilevel data analysis and visualization. We used the ggplot2 package to create bar charts, which respectively showed the top 15 common targets associated with the most compounds, the number of common targets each herb acted upon, and the top 15 compounds that acted on the most common targets. We used the pheatmap package to draw cluster heatmaps, which demonstrated the strength of the association between the main herbs and the key common targets. Finally, we used the UpSetR package to draw UpSet diagrams, which precisely displayed the complex intersection of shared targets among different herb combinations.

### 2.8. Statistical Analysis

All statistical analyses were performed in R software (Version 4.0). scRNA‐seq data analysis used the Seurat package, dimensionality reduction visualization employed UMAP and t‐SNE algorithms [16, 17], and cell clustering used the Leiden algorithm. Developmental trajectory analysis employed the Monocle package, and functional enrichment analysis used the clusterProfiler package. Differential gene expression analysis employed the Wilcoxon rank‐sum test or negative binomial distribution test, with *p*‐values less than 0.05 considered statistically significant. All analysis results were presented through various visualization methods, including scatter plots, violin plots, heatmaps, and network diagrams, ensuring result intuition and interpretability.

## 3. Results

### 3.1. Single‐Cell Transcriptomic Data Quality Control and Dimensionality Reduction Analysis in Pediatric Asthma

**FIGURE 1:**
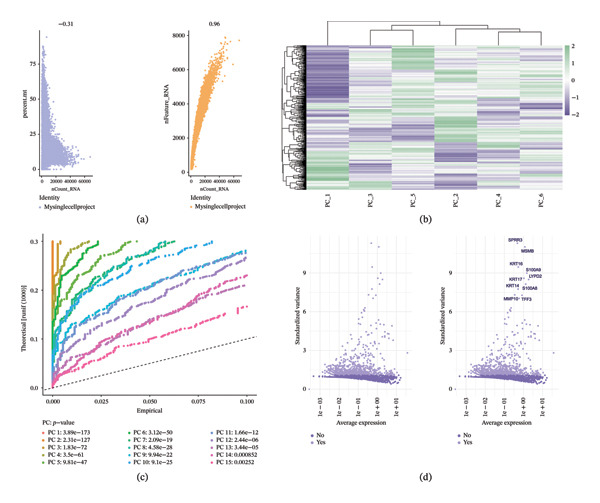
Quality control and dimensionality reduction analysis of single‐cell RNA sequencing data in pediatric asthma. (a) RNA count feature distribution and dataset correlation analysis, showing data quality and batch consistency; correlation coefficients are reported as Pearson’s r. (b) Highly variable gene identification, annotating key epithelial and inflammation‐related genes. (c) Gene expression hierarchical clustering heatmap, displaying expression pattern differences among different cell populations. (d) Principal component analysis results, showing the contribution and statistical significance of the first 15 principal components.

Quality control analysis of scRNA‐seq data demonstrated excellent data quality characteristics. Figure 1(a) shows the distribution patterns of RNA count features, with the left scatter plot displaying a negative correlation between the number of detected genes per cell and total RNA counts (correlation coefficient −0.31), while the right plot shows high consistency between the two datasets (correlation coefficient 0.96), indicating effective control of batch effects. This distribution pattern conforms to typical single‐cell data characteristics, establishing a reliable data foundation for subsequent analyses. Figure 1(b) identifies key genes with high biological variability through the relationship between average expression and standardized variance. In the region of high expression and high variability, multiple important genes related to epithelial cell differentiation and inflammatory responses were detected, including keratin family genes KRT16, KRT17, KRT14, and inflammation‐related genes SPRR3, MSMB, among others. The identification of these highly variable genes provides a molecular basis for understanding cellular heterogeneity and functional differences in asthma pathological processes.

The hierarchical clustering heatmap (Figure 1(c)) reveals gene expression difference patterns among different cell populations. The heatmap displays distinct clustering structures, with different cell types exhibiting specific gene expression profiles, where green represents high expression and purple represents low expression. This clustering pattern reflects the functional specialization of different cell types in asthmatic airways, providing important evidence for cell type annotation and functional analysis. PCA results (Figure 1(d)) show that the first 15 principal components effectively capture the main variational information in the data. The number of PCs retained was determined using elbow plot inspection and variance‐explained patterns, and the variance contribution of the top PCs is shown in Figure 1(d). All principal components demonstrate significant statistical significance (all *p*‐values < 0.001), with the first three principal components contributing most prominently. This dimensionality reduction result provides an optimized data structure for subsequent cell clustering analysis and trajectory inference, facilitating the identification of asthma‐related cell state transitions and developmental trajectories.

### 3.2. Single‐Cell Atlas Construction and Cell Type Identification of Pediatric Asthma Airway Epithelial Cells

**FIGURE 2:**
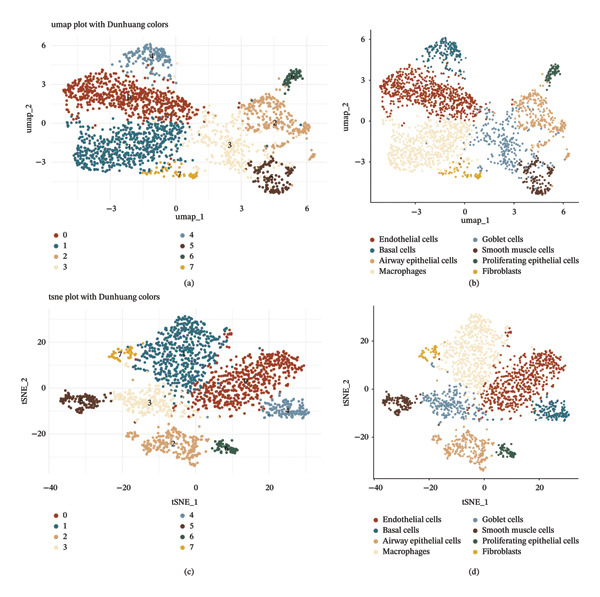
Single‐cell atlas and functional annotation of airway epithelial cells in pediatric cough‐variant asthma. (a–b) UMAP plots of cell clustering based on gene expression similarity, with different colors representing distinct cell populations. (c–d) Cell type annotation based on marker gene expression, identifying seven major airway epithelial cell subtypes including eosinophil cells, basal cells, ciliated cells, goblet cells, club cells, ionocyte cells, and deuterosomal cells.

Through UMAP and t‐SNE dimensionality reduction, we constructed a two‐dimensional map of airway tissue‐derived single cells from pediatric cough‐variant asthma samples (Figures 2(a) and 2(b)). Clustering based on transcriptomic similarity separated the cells into eight major compartments, indicating a stable global structure of the dataset rather than diffuse, overlapping signals. Based on canonical marker genes, these clusters were annotated as endothelial cells, fibroblasts, smooth muscle cells, macrophages, and four epithelial‐related populations (airway epithelial cells, basal cells, goblet cells, and proliferating epithelial cells) (Figures 2(c) and 2(d)). Marker genes used for annotation were identified by differential expression testing (Wilcoxon rank‐sum test with Benjamini–Hochberg correction), and markers with adjusted *p* < 0.05 were considered statistically significant. Notably, this atlas captures both epithelial and nonepithelial components of the airway microenvironment, allowing epithelial dysfunction to be interpreted alongside immune and stromal programs that are tightly linked to asthma‐related inflammation and remodeling. Within the epithelial compartment, the concurrent presence of basal, goblet, and proliferating epithelial states supports an active epithelial turnover/repair context and mucus‐related specialization, which is consistent with chronic inflammatory perturbation at the tissue level. Meanwhile, the colocalization of macrophages with smooth muscle and fibroblast compartments in the same dataset provides a framework for discussing inflammatory–remodeling coupling, rather than treating epithelial changes in isolation.

### 3.3. Key Gene Expression Characteristics Analysis in Pediatric Asthma Airway Epithelial Cells

Through single‐cell gene expression analysis, multiple key genes with important functions in pediatric asthma airway epithelial cells were identified. Among these, MALAT1, as a long noncoding RNA, is expressed in almost all cell types, demonstrating its ubiquity as a housekeeping gene (Figure 3(a)). KRT16 shows a preferential distribution across epithelial‐associated compartments, with enriched signal in basal lineage–related areas on the embedding, reflecting the epithelial activation/repair context of the airway mucosa. CXCL5 (C‐X‐C motif chemokine ligand 5), as a chemokine, exhibits a localized expression pattern in restricted cell compartments rather than a diffuse background, suggesting cell type‐dependent inflammatory recruitment signaling within the tissue microenvironment. Inflammation and tissue remodeling–related genes display differential expression patterns across different cell subpopulations. MMP10 (Matrix Metalloproteinase 10) is expressed in specific cell populations, participating in extracellular matrix degradation and airway remodeling processes. ADAM12, as a disintegrin and metalloproteinase, shows expression in certain cells, suggesting its role in cell adhesion and signal transduction. The specific expression pattern of IGFBP7 indicates its potential involvement in cell proliferation and differentiation regulation, which is significant in asthmatic airway epithelial repair processes.

FIGURE 3Single‐cell expression analysis of key genes in pediatric asthma airway epithelial cells. (a) UMAP feature plots showing the spatial expression distribution of representative genes across the airway single‐cell embedding, where purple/blue dots indicate cells with higher expression and gray dots represent cells with low or undetectable expression. (b) DotPlot summarizing cell type–level localization of key genes, with dot size representing the percentage of expressing cells and color indicating scaled average expression. Genes were organized into three mechanistically relevant signatures, including homeostasis/barrier defense, CXCL5‐linked inflammatory recruitment, and repair–remodeling (KRT16–MMP10/ADAM12 axis), enabling interpretation of gene expression patterns within epithelial–immune remodeling contexts. Genes shown were selected from cluster‐level differential expression analysis using an adjusted *p* value < 0.05 threshold after multiple‐testing correction.

**Figure:**
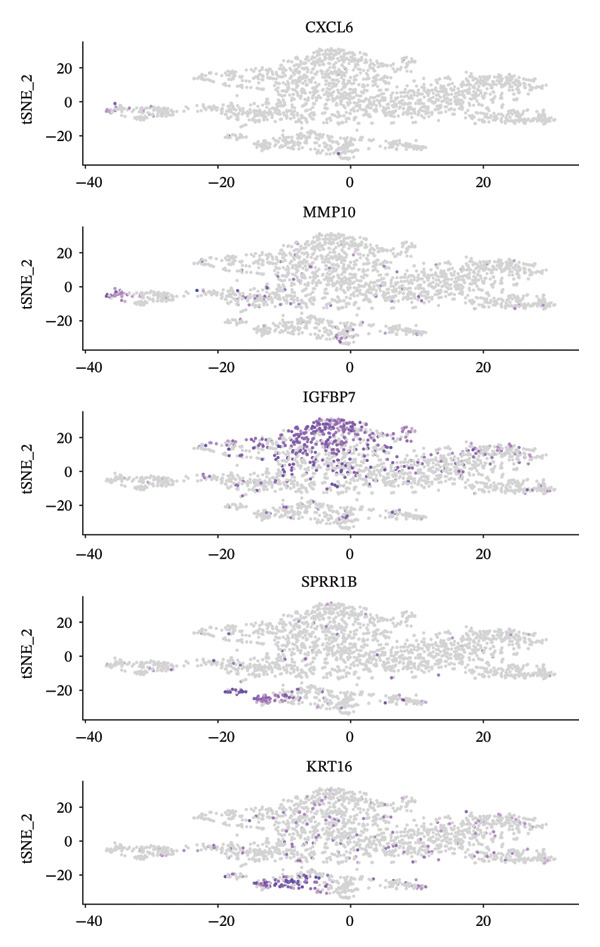
(a)

**Figure:**
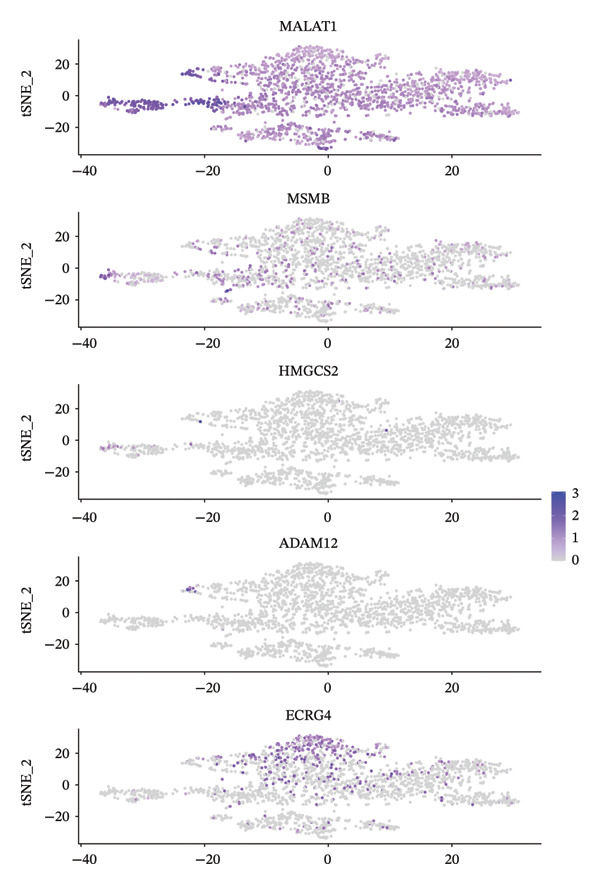
(b)

The expression of genes such as HMGCS2, MSMB, SPRR1B, and ECRG4 in specific cell subpopulations reflects the metabolic characteristics and functional specialization of different cell types. HMGCS2 participates in cholesterol synthesis pathways, and its expression in certain cells may be related to cell membrane composition and signal transduction. To summarize localization at the cell type level, DotPlot visualization was used to jointly display average expression and the proportion of expressing cells (Figure 3(b)), allowing the above genes to be interpreted as three broad functional signatures, including homeostasis/barrier defense, chemokine‐linked inflammatory recruitment, and repair–remodeling programs. The differential expression patterns of these genes provide a molecular basis for understanding the functional heterogeneity and pathophysiological processes of asthmatic airway epithelial cells, helping to identify potential therapeutic targets and biomarkers (Figure 3). The genes shown in Figure 3 were selected as representative markers based on cluster‐level differential expression results, using an adjusted *p* value of < 0.05 (multiple‐testing corrected) threshold.

### 3.4. Developmental Trajectory and Functional Differentiation Analysis of Pediatric Asthma Airway Epithelial Cells

**FIGURE 4:**
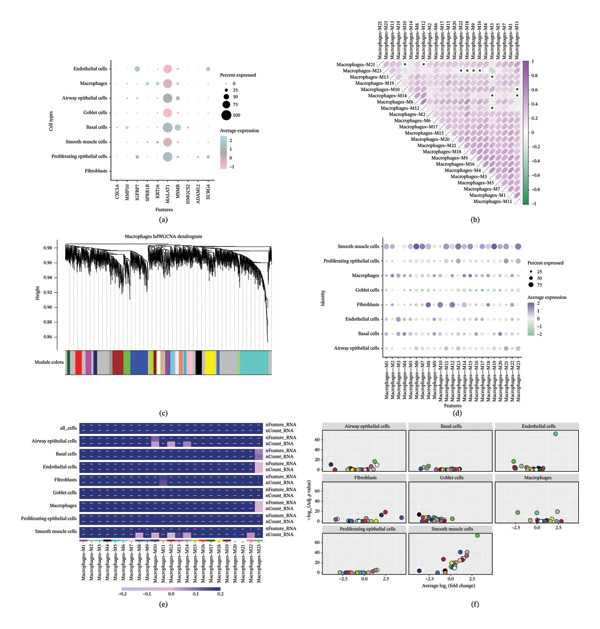
Developmental trajectory and gene expression dynamics analysis of pediatric asthma airway epithelial cells. (a) Dot plot of cell type–specific marker gene expression, with dot size representing the proportion of expressing cells and color intensity representing average expression level; marker genes were identified by differential expression testing using the Wilcoxon rank‐sum test with Benjamini–Hochberg correction (adjusted *p* < 0.05). (b) Cell developmental trajectory reconstructed using Monocle algorithm, showing differentiation pathways from basal cells to various differentiated cell types. (c–d) Gene expression heatmaps along developmental trajectory and correlation analysis between cell types. (e) Cell distribution and state transition patterns in trajectory space. (f) Expression heatmap analysis of functionally related gene modules.

Through differential gene expression analysis (Figure 4(a)), characteristic marker genes for each cell subtype were successfully identified. The dot plot displays the expression percentage and average expression intensity of different genes across various cell types, where the dot size represents the proportion of cells expressing the gene and color intensity reflects the average expression level. Marker genes were defined using differential expression testing (Wilcoxon rank‐sum test) with Benjamini–Hochberg correction, and adjusted *p* < 0.05 was considered statistically significant. Microvascular endothelial cells, ciliated cells, basal cells, goblet cells, and others each exhibit unique gene expression characteristics, providing reliable molecular evidence for cell type identification and functional annotation. Pseudotime trajectory analysis (Figure 4(b)) revealed the developmental differentiation process of airway epithelial cells. The cell developmental trajectory reconstructed using the Monocle algorithm shows that basal cells, as the stem cell population, are located at the starting point of the trajectory and subsequently differentiate into various terminally differentiated cell types. The colored bands below the trajectory plot represent the distribution of different cell types along the developmental trajectory, demonstrating the continuous differentiation process from multipotent stem cells to specialized cells, which may be aberrant under asthmatic pathological conditions. Heatmap analyses (Figures 4(c) and 4(d)) display the dynamic changes in gene expression along the developmental trajectory. Figure 4(c) shows the expression changes of different gene modules along the pseudotime axis, with purple indicating high expression and white indicating low expression. These gene modules reflect key molecular events during cell differentiation. Figure 4(d) further displays the correlation of gene expression between different cell types, with dot size and color intensity representing the strength of expression correlation, providing important information for understanding functional relationships between cells. Trajectory analysis results (Figure 4(e)) show the distribution of cells in two‐dimensional space and their state changes along the developmental trajectory. Different colored dots represent cells at different differentiation stages, and trajectory lines show possible pathways of cell state transitions. This analysis helps identify key nodes of abnormal airway epithelial cell differentiation under asthmatic conditions. Functional enrichment analysis (Figure 4(f)) further reveals gene modules related to cell differentiation, inflammatory responses, and airway remodeling, providing systematic molecular insights into asthma pathogenesis. Only enriched terms/pathways meeting FDR < 0.05 after multiple‐testing correction were considered statistically significant and reported.

### 3.5. Macrophage Subpopulation Heterogeneity and Functional Program Stratification in Pediatric Asthma Airways

High‐resolution analysis of the macrophage compartment resolved 23 distinct macrophage‐associated transcriptional programs (M1–M23), summarized as gene coexpression modules (Figures 5(a) and 5(b)). In Figure 5(a), each module is characterized by a set of highly connected hub genes ranked by kME (module membership), indicating that the macrophage signal is organized into multiple internally coherent gene programs rather than a single uniform activation state. Notably, several modules are dominated by housekeeping or ribosome‐related hubs, whereas others are enriched for stress response, antigen‐presentation, or signaling‐related hubs, suggesting heterogeneous functional bias across macrophage states. Figure 5(b) further maps the module‐level activity (eigengene/module score) onto the macrophage embedding, showing that different modules peak in distinct but partially continuous regions, consistent with a mixture of discrete states and intermediate transitions within the airway immune niche. Taken together, this module‐based stratification provides a function‐oriented framework to interpret macrophage heterogeneity in pediatric asthma and supports prioritization of specific macrophage programs for downstream validation, instead of relying on an oversimplified M1/M2 dichotomy.

FIGURE 5Module‐based stratification of macrophage transcriptional programs in pediatric cough‐variant asthma airways. (a) Identification of 23 macrophage‐associated gene coexpression modules (M1–M23). For each module, representative hub genes are shown and ranked by kME (module membership), reflecting the internal connectivity and coherence of the module‐level program. (b) Spatial mapping of module activity across the macrophage embedding. Each panel displays the distribution of the corresponding module score/eigengene signal, highlighting regions with higher module activity and suggesting heterogeneous macrophage programs with partially continuous transitions rather than a single uniform activation state. Collectively, these results support a function‐oriented interpretation of macrophage heterogeneity and provide a rational basis for prioritizing macrophage programs for downstream validation. Functional enrichment analysis and pathway network resolution of pediatric asthma airway cells.

**Figure:**
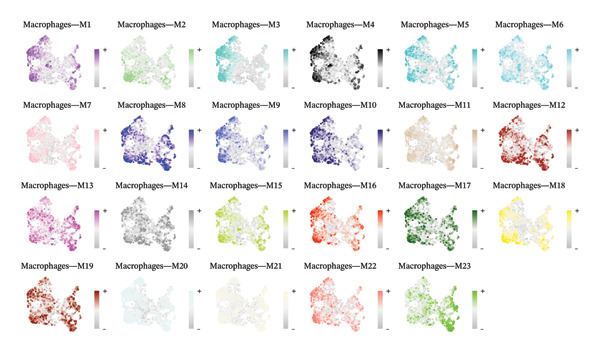
(a)

**Figure:**
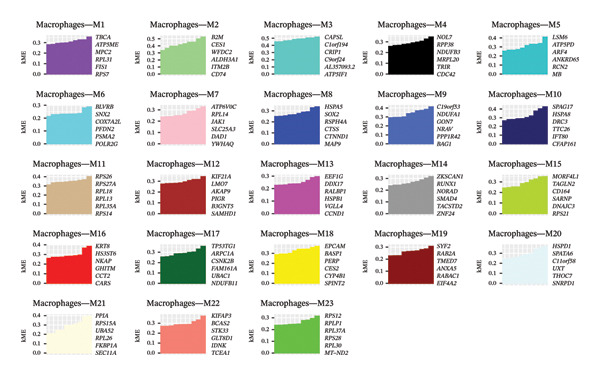
(b)

Through systematic functional enrichment analysis, the molecular functional characteristics of pediatric asthma airway cells were comprehensively analyzed. Biological process enrichment analysis (Figure 6(a)) shows that differentially expressed genes are mainly enriched in key biological processes such as inflammatory responses, immune system processes, cell proliferation, and differentiation. Among these, immune response–related processes showed the highest enrichment significance, reflecting the essential characteristics of asthma as an immune‐mediated disease. Cellular component analysis (Figure 6(b)) revealed that these genes are mainly localized to important cellular structures such as cell membranes, cytoplasm, and mitochondria, suggesting the important roles of cell membrane receptor signaling and mitochondrial metabolism in asthma pathogenesis. Molecular function enrichment analysis (Figure 6(c)) identified key molecular functional categories, including protein binding, enzyme activity regulation, and transcription factor activity. The enrichment of these molecular functions indicates that complex molecular regulatory networks exist in asthmatic airway cells, involving multiple levels such as protein interactions, enzymatic reaction regulation, and gene transcriptional control. GO enrichment results (Figure 6(d)) further quantified the enrichment degree of each functional category, with inflammation‐related functions and cellular signaling functions showing the most significant enrichment, confirming the central position of these processes in asthma pathological mechanisms. The circular network diagram (Figure 6(e)) displays the complex associations between different functional modules, with connecting lines of different colors representing the strength of interfunctional relationships. This network structure reveals the systemic characteristics of asthma‐related gene functions, showing close functional coordination relationships among multiple signaling pathways and biological processes. Pathway enrichment analysis (Figure 6(f)) identified multiple classical signaling pathways closely related to asthma pathogenesis, including inflammatory signaling pathways, immune response pathways, and apoptosis pathways, providing a systematic molecular atlas for understanding asthma molecular mechanisms and searching for potential therapeutic targets. GO and KEGG enrichment significance was evaluated with multiple‐testing correction, and only terms/pathways meeting an FDR‐adjusted *p* value of < 0.05 were considered statistically significant and presented.

FIGURE 6Functional enrichment analysis and pathway network resolution of pediatric asthma airway cells. (a–c) GO functional enrichment analysis results, showing enrichment of biological processes, cellular components, and molecular functions, respectively, with dot size representing the number of enriched genes and color intensity representing significance level; enrichment terms were considered significant at FDR < 0.05 after multiple‐testing correction. (d) Quantitative results of GO enrichment analysis, with bar charts showing enrichment scores for each functional category. (e) Interfunctional module association network diagram, displaying interactions between different biological processes and molecular functions. (f) Signal pathway enrichment analysis results, identifying key regulatory pathways related to asthma; only pathways meeting FDR‐adjusted *p* value < 0.05 are reported.

**Figure:**
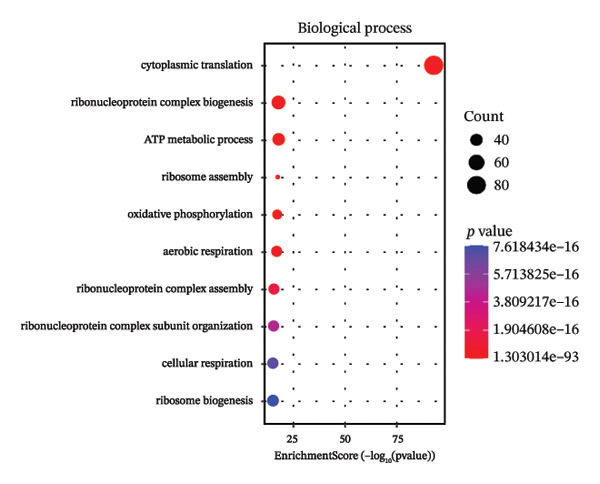
(a)

**Figure:**
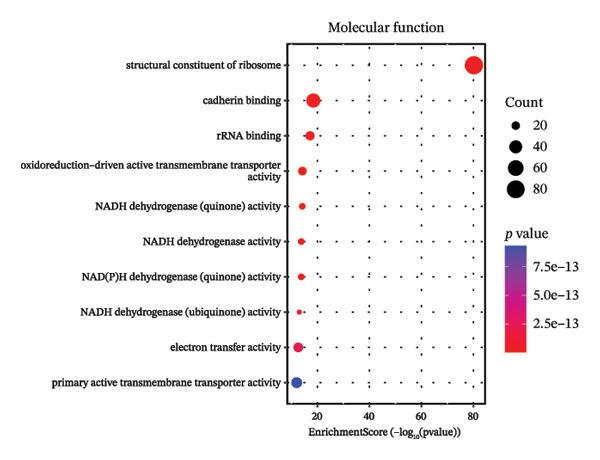
(b)

**Figure:**
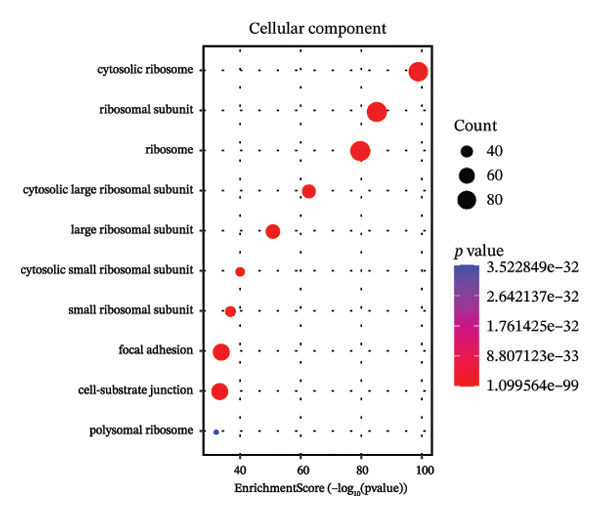
(c)

**Figure:**
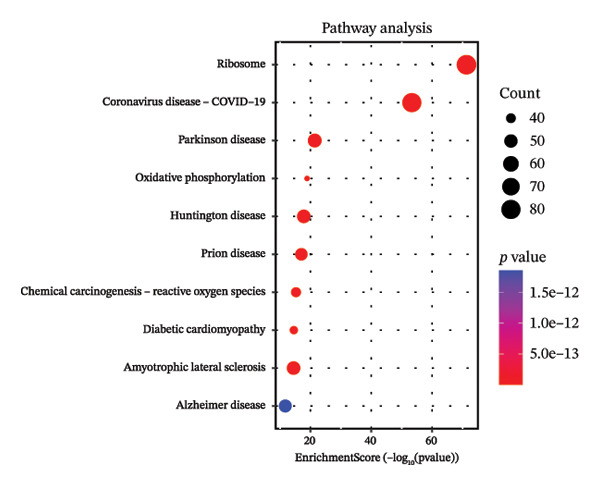
(d)

**Figure:**
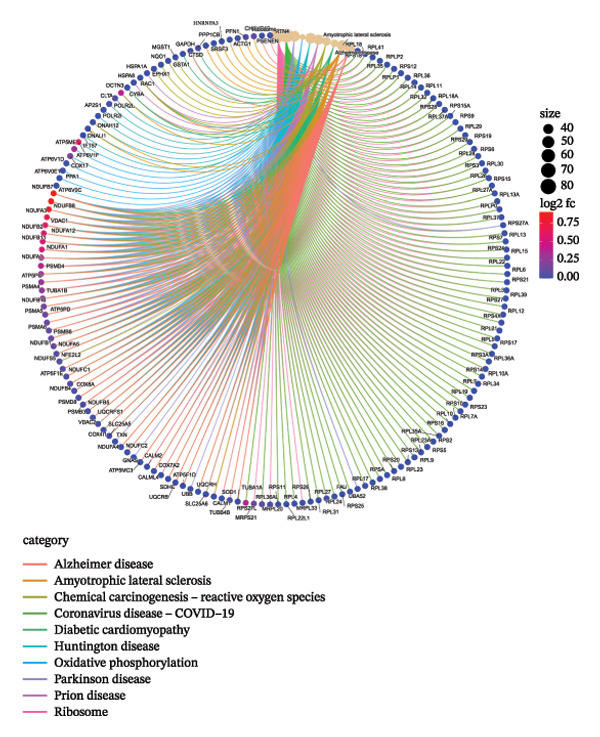
(e)

**Figure:**
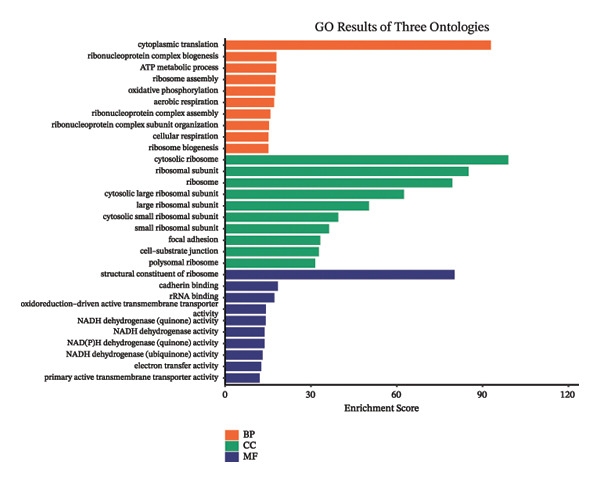
(f)

### 3.6. Network Pharmacology Analysis of XQLT in Asthma

**FIGURE 7:**
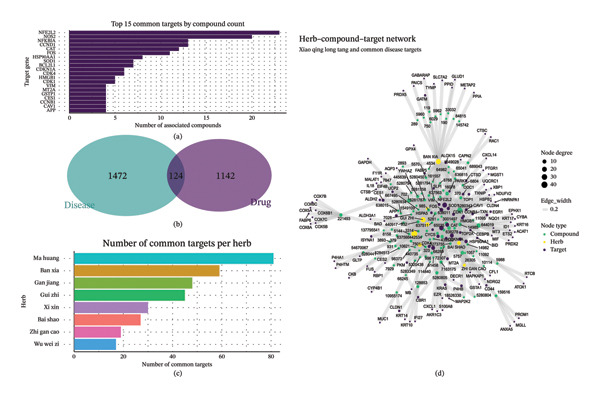
Network pharmacology analysis of XQLT on asthma targets (network pharmacology analysis of XQLT on asthma targets). (a) Bar chart ranking the top 15 common targets by the number of compounds, with the abscissa representing the target genes and the ordinate representing the number of compounds associated with each target. (b) Venn diagram of drug targets and disease targets, showing the number of intersections between the two. (c) Bar chart of the number of common targets for each herb; (d) Interaction network diagram of “herb–compound–target,” where yellow diamonds represent herbs, purple dots represent compounds, and green dots represent common targets. The size of the nodes is proportional to the degree (number of connections) of the nodes, and the width of the edges represents the strength of the interaction.

Network pharmacology analysis was conducted to characterize the multitarget regulatory features of XQLT decoction in asthma. A total of 1266 putative drug targets and 1596 asthma‐related disease targets were retrieved, with 124 overlapping targets identified as potential therapeutic nodes (Figure 7(b)). The top‐ranked common targets by compound count (Figure 7(a)), including NFE2L2, NOS2, NFKBIA, CCND1, and CAT, were mainly enriched in inflammatory signaling, oxidative stress response, and stress‐related transcriptional regulation. Herb‐level analysis showed that Ma Huang and Ban Xia contributed the largest number of common targets (Figure 7(c)). The herb–compound–target network (Figure 7(d)) further illustrated the multicomponent and multitarget structure of the formula, highlighting highly connected hubs such as NFE2L2, HSP90AA1, and FOS. Rather than indicating cell‐specific effects, these hub targets converge on inflammation and stress response programs, which align with the dominant immune‐ and remodeling‐related processes identified in the single‐cell analysis.

### 3.7. Analysis of Key Components and Target Interaction Patterns of XQLT

To further delineate the cooperative features of XQLT, target interaction patterns among individual herbs were analyzed. The clustered heatmap (Figure 8(a)) revealed that multiple herbs, particularly Ban Xia, Gan Jiang, and Ma Huang, shared strong associations with inflammation‐ and oxidative stress–related targets such as NFE2L2, NOS2, and NFKBIA, whereas Bai Shao exhibited a distinct interaction profile. Analysis of active compounds (Figure 8(b)) identified several highly connected molecules, including quercetin, luteolin, and β‐sitosterol, suggesting their central roles in mediating multitarget effects. The UpSet analysis (Figure 8(c)) further demonstrated extensive target overlap among different herbs, supporting a synergistic, program‐oriented mode of action. Collectively, these results indicate that XQLT preferentially modulates inflammation‐ and stress‐associated regulatory axes, providing a mechanistic complement to the immune and epithelial remodeling programs highlighted by the single‐cell transcriptomic analysis.

FIGURE 8Analysis of key components and target interactions of XQLT (analysis of key components and target interactions of XQLT). (a) Heatmap showing the relationship between the main herbs and the key common targets, with purple indicating strong correlation and white indicating weak correlation or no correlation. (b) Bar chart ranking the top 15 compounds (represented by PubChem CID) by the number of common targets they act upon. (c) UpSet diagram of common targets among the herbs. The left bar chart shows the total number of targets affected by each herb, the upper bar chart shows the number of unique or shared targets among different herb combinations, and the dot matrix below represents the herb combinations that are involved in the intersection.

**Figure:**
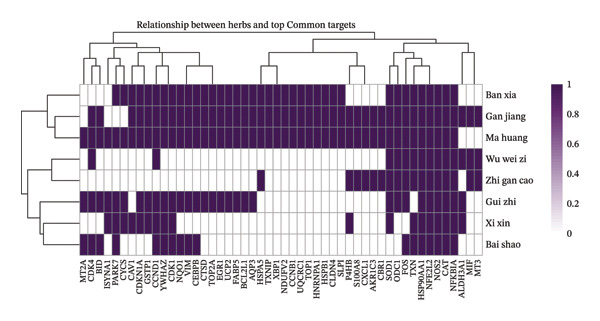
(a)

**Figure:**
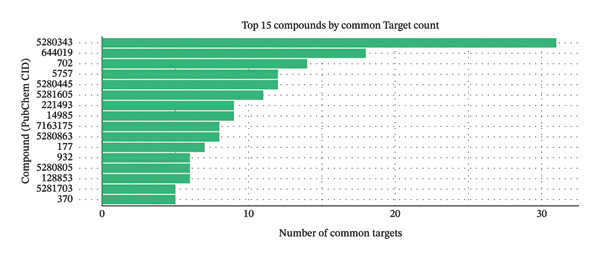
(b)

**Figure:**
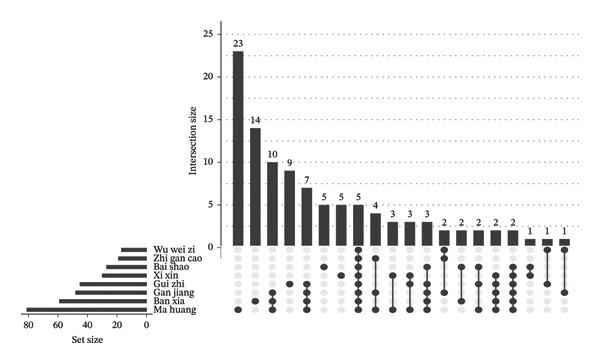
(c)

## 4. Discussion

Our comprehensive single‐cell analysis reveals the remarkable cellular diversity within pediatric asthmatic airways, identifying seven major epithelial cell subtypes with distinct molecular signatures and functional specializations. The identification of rare cell populations such as ionocytes and deuterosomal cells significantly expands our understanding of airway epithelial complexity and suggests previously unrecognized roles for these cells in asthma pathogenesis [18]. The predominance of basal cells in our dataset reflects their crucial role as epithelial stem cells responsible for airway repair and regeneration [19]. In our single‐cell atlas, basal cells occupy transcriptional states enriched for stress‐ and proliferation‐associated programs, and in asthmatic conditions, basal cells may undergo aberrant activation and differentiation, contributing to pathological airway remodeling. The coexistence of ciliated cells and goblet cells in varying proportions highlights the functional imbalance characteristic of asthmatic airways, where impaired mucociliary clearance coincides with excessive mucus production.

Beyond cell type annotation, the single‐cell atlas adds functional insight into airway pathology in pediatric cough‐variant asthma. The spatial arrangement of basal, goblet, and ciliated epithelial populations integrates epithelial regeneration, mucus‐related activity, and barrier‐associated functions within a unified tissue context, and this organization is supported by compartment‐specific enrichment of inflammation‐ and remodeling‐related pathways in our data, implying that chronic inflammatory exposure may alter epithelial homeostasis and favor remodeling‐associated differentiation patterns [20]. In parallel, pathway enrichment signals related to inflammation and tissue remodeling consistently localize to specific epithelial compartments, supporting the biological relevance of these cell subsets and indicating that the observed heterogeneity reflects disease‐associated functional programs rather than purely descriptive classification [21].

The differential expression of keratin family genes, particularly KRT16, KRT17, and KRT14, provides crucial insights into the structural alterations occurring in asthmatic airway epithelium. In our dataset, KRT16 shows preferential expression in basal and proliferative epithelial states, representing an important marker of epithelial activation and proliferation. In the context of asthma, elevated KRT16 expression may indicate enhanced epithelial turnover and repair responses following inflammatory injury. KRT17 has been implicated in epithelial barrier function and inflammatory signaling [22, 23], while KRT14 serves as a classical basal cell marker essential for maintaining epithelial integrity [24–26].

The coordinated expression of these keratin genes in specific cell populations suggests their roles in maintaining cytoskeletal architecture during the dynamic processes of epithelial repair and remodeling that characterize asthmatic airways. Importantly, the restriction of KRT16/17 expression to defined epithelial states in our analysis supports a repair‐biased, stress‐responsive epithelial program rather than uniform epithelial activation, and these cell type–specific expression patterns may serve as valuable biomarkers for disease monitoring. Notably, this keratin‐restricted pattern also argues against a nonspecific “whole‐epithelium activation” interpretation and instead points to discrete basal/proliferative compartments that may disproportionately contribute to remodeling‐prone repair.

CXCL5 emerges as a critical mediator of inflammatory cell recruitment in asthmatic airways. As a potent neutrophil chemoattractant, CXCL5 plays essential roles in orchestrating innate immune responses and perpetuating airway inflammation [27–29]. Its specific expression in certain epithelial cell populations suggests that these cells serve as important sources of proinflammatory signals, contributing to the recruitment and activation of immune cells within the asthmatic microenvironment. Consistent with this observation, elevated CXCL5 expression has been linked to increased neutrophilic inflammation in more severe asthma phenotypes, indicating that epithelial CXCL5‐associated programs may contribute to sustained airway immune activation and represent potential targets for anti‐inflammatory intervention. Importantly, the nonuniform localization of CXCL5 in our dataset supports a compartmentalized epithelial–immune signaling model, in which only specific epithelial states function as chemokine “sources” driving inflammatory recruitment, rather than CXCL5 being a diffuse marker of global inflammation.

The expression of MMP10 and ADAM12 in specific cell populations highlights the active tissue remodeling processes occurring in asthmatic airways. In our analysis, MMP10 and ADAM12 are enriched in defined epithelial subsets rather than uniformly expressed, indicating ongoing extracellular matrix turnover linked to specific cellular states. MMP10, also known as stromelysin‐2, participates in extracellular matrix degradation and has been implicated in airway remodeling processes including subepithelial fibrosis and smooth muscle hyperplasia. ADAM12, a member of the ADAM (a disintegrin and metalloproteinase) family, functions in cell adhesion, migration, and signaling. Its expression in asthmatic airway epithelium suggests roles in epithelial–mesenchymal interactions and cellular communication during airway remodeling [30, 31]. Together, the cell state–specific expression of MMP10 and ADAM12 supports the presence of persistent, program‐driven remodeling activity in pediatric asthma rather than transient injury responses.

The 23 macrophage subpopulations observed in this study indicate a multistate organization of airway macrophages in pediatric asthma, encompassing inflammatory, repair‐associated, and intermediate transcriptional programs within the same tissue environment. Rather than representing discrete cell types, these macrophage programs coexist along a continuum and are spatially and transcriptionally aligned with epithelial remodeling and inflammatory niches identified in our atlas. Importantly, this program‐level framework also helps contextualize the potential action of XQLT, a classical formula widely used for asthma‐related cough and wheezing, which has been reported to modulate airway inflammation and oxidative stress–related responses. In line with this, our network pharmacology provides a complementary, program‐level perspective, as its core targets are mainly linked to inflammation, oxidative stress, and stress‐responsive regulation, which parallel the immune activation and remodeling processes identified by single‐cell analysis. Integrating these findings at the level of shared biological processes allows predicted molecular targets to be interpreted within macrophage‐associated and epithelial remodeling contexts, thereby reinforcing a program‐oriented, rather than cell type–restricted, mechanistic framework for pediatric asthma.

## 5. Limitations

Several limitations should be acknowledged in interpreting these results. The cross‐sectional nature of the study limits our ability to assess temporal changes in cellular states. Additionally, the focus on epithelial tissues may not capture the full complexity of immune‐epithelial interactions that characterize asthmatic airways.

## 6. Conclusion

This comprehensive single‐cell transcriptomic analysis of pediatric asthma airway epithelium reveals unprecedented cellular heterogeneity and provides novel insights into disease pathogenesis. We successfully constructed a detailed cellular atlas identifying seven major epithelial cell subtypes and 23 distinct macrophage subpopulations, each with unique molecular signatures and functional specializations.

## Funding

This study was supported by the National Key R&D Program of China (No. 2024YFC3506002), titled “Clinical Study and Efficacy Evaluation System Construction of National TCM Master′s Experience Formula for Pediatric Mycoplasma Pneumoniae Pneumonia.”; Shandong Province “Qilu Biancang Traditional Chinese Medicine Talent” Cultivation Project, Project No. Lu Wei TCM Science and Education Document [2025] No. 2; “Young Traditional Chinese Medicine Physician” Cultivation Project of the Affiliated Hospital of Shandong University of Traditional Chinese Medicine, Project No. Sheng Zhong Ren Zi [2024] No. 60; and Shandong Province Traditional Chinese Medicine Science and Technology Plan Project: Mechanism Study of Linggan Wuwei Jiangxin Decoction in Regulating Airway Hyperresponsiveness in Cough Variant Asthma via Inhibiting the TRPV1‐CGRP Neurogenic Inflammatory Pathway, Project No. M20251706.

## Ethics Statement

The authors have nothing to report.

## Conflicts of Interest

The authors declare no conflicts of interest.

## Data Availability

The data that support the findings of this study are available from the corresponding authors upon reasonable request.

## References

[1] Alrashide A. S. , Alessa M. Q. , Elhefny M. et al., Assessment of Public Awareness and Prevalence of Asthma Comorbidities in Al-Qunfudhah, Saudi Arabia, Cureus. (2025) 17, no. 6, 10.7759/cureus.85389. PMC12228079 40621261

[2] Lu Z. , Yang H. , Yan M. et al., The Effects of Different Follow-Up Management on Asthma Control: A Prospective Cohort Study, BMC Pulmonary Medicine. (2025) 25, no. 1, 10.1186/s12890-025-03380-x. PMC12228337 40615808

[3] Lian D. , Lin C. Y. , Xie M. L. et al., Integrative Bioinformatics Analysis of Pyroptosis-Related Genes and Immune Infiltration Patterns in Childhood Asthma, Frontiers in Genetics. (2025) 16, 10.3389/fgene.2025.1557709. PMC12226291 40620703

[4] Breidenbach J. D. , French B. W. , Shrestha U. et al., Acute Exposure to Aerosolized Nanoplastics Modulates Redox-Linked Immune Responses in Human Airway Epithelium, Antioxidants (Basel). (2025) 14, no. 4, 10.3390/antiox14040xxx. PMC12024294 40298680

[5] Ma L. , Thapa B. R. , Le Suer J. A. et al., Author Correction: Life-Long Functional Regeneration of in Vivo Airway Epithelium by the Engraftment of Airway Basal Stem Cells, Nature Protocols. (2025) 10.1038/s41596-025-xxxxx-x. 40164751

[6] Aggarwal S. , Chakraborty A. , Singh V. , Lory S. , Karalis K. , and Rahme L. G. , Revealing the Impact of Pseudomonas aeruginosa Quorum Sensing Molecule 2’-Aminoacetophenone on Human Bronchial-Airway Epithelium and Pulmonary Endothelium Using a Human Airway-on-a-Chip, 2025, bioRxiv. 10.3389/fimmu.2025.1592597 PMC12305196 40735328

[7] Lamba R. , Paguntalan A. M. , Petrov P. B. , Naba A. , and Izzi V. , MatriCom: A scRNA-Seq Data Mining Tool to Infer ECM-ECM and Cell-ECM Communication Systems, Journal of Cell Science. (2025) 138, 10.1242/jcs.xxxxxx. PMC12276803 40501363

[8] Shu Z. Q. , Ren Y. X. , Long Q. H. , Wang H. B. , and Yu Z. T. , scGANSL: Graph Attention Network With Subspace Learning for scRNA-Seq Data Clustering, Journal of Chemical Information and Modeling. (2025) 65, no. 12, 6367–6381, 10.1021/acs.jcim.5c01234. 40468846

[9] Wei X. , Ma W. J. , Wu Z. J. , and Wu H. , TORC: Target-Oriented Reference Construction for Supervised Cell-Type Identification in scRNA-Seq, Genome Biology. (2025) 26, no. 1, 10.1186/s13059-025-03450-x. PMC12150436 40495172

[10] Sun Z. F. , Zhou R. , Ning C. H. et al., scRNA-Seq and Bulk-Seq Identify Novel Markers Associated With Calcium Regulation and Immunomodulation in Acute Pancreatitis, Pancreas. (2025) 54, no. 6, e512–e523, 10.1097/MPA.0000000000002456. 40536508

[11] Liu H. , Zhang Y. S. , and Ning S. B. , ScRNA-Seq Combined With ATAC-Seq Analysis to Explore the Metabolic Balance Mechanism of CCl4-Induced Liver Inflammatory Injury, Frontiers in Immunology. (2025) 16, 10.3389/fimmu.2025.1600685. PMC12206626 40589742

[12] Huang P. J. , Tsai F. Y. , Wu Y. J. et al., ShinySC: An R/Shiny-Based Desktop Application for Seamless Analysis of scRNA-Seq Data, Biomedical Journal. (2025) 49, no. 1, 10.1016/j.bj.2025.100885. PMC12860678 40609640

[13] Zhan Z. Y. , Chen H. L. , Wang T. , Wang T. T. , and Chen X. G. , Identification of lncRNA-miRNA-mRNA ceRNA Axes and KEGG Pathways Related to Uveal Melanoma Metastasis, Technology and Health Care. (2025) 33, no. 3, 1522–1531, 10.3233/THC-241234. 39973883

[14] Kanehisa M. , Furumichi M. , Sato Y. , Matsuura Y. , and Ishiguro-Watanabe M. , KEGG: Biological Systems Database as a Model of the Real World, Nucleic Acids Research. (2025) 53, no. D1, D672–D677, 10.1093/nar/gkae1234. 39417505 PMC11701520

[15] Okamura R. , Aoyama R. , Tsunoda S. et al., Management Challenges and the Role of Adjuvant Chemotherapy in Remnant Gastric Cancer: An Analysis of 313 Patients From the KEGG Multicenter Observational Study, Gastric Cancer. (2024) 27, no. 6, 1302–1310, 10.1007/s10120-024-01567-x. 39115631

[16] Lee Y. J. , Jeong C. W. , and Kim H. J. , Forensic Feature Extraction of Document Paper Using Periodic Marks: PCA and t-SNE for Manufacturer Discrimination and Document Dating, Forensic Science International. (2025) 367, 10.1016/j.forsciint.2025.112348. 39733694

[17] Marques J. G. , de Carvalho B. M. , Guedes L. A. , and Da Costa-Abreu M. , Pattern Recognition in SARS Cases: Insights From t-SNE and k-Means Clustering Applied to COVID-19 Symptomatology, Frontiers in Artificial Intelligence. (2025) 8, 10.3389/frai.2025.1536486. PMC11983552 40212085

[18] Chen L. , Hoefel G. A. , Pathinayake P. S. et al., Inflammation-Induced Loss of CFTR-Expressing Airway Ionocytes in Non-Eosinophilic Asthma, Respirology. (2025) 30, no. 1, 25–40, 10.1111/resp.14890. 39358991 PMC11688627

[19] Rouhani M. J. , Janes S. M. , and Kim C. F. , Epithelial Stem and Progenitor Cells of the Upper Airway, Cells & Development. (2024) 177, 10.1016/j.cdev.2024.203905. 38355015

[20] Liu Z. Y. , Zheng Q. , Li Z. B. et al., Epithelial Stem Cells From Human Small Bronchi Offer a Potential for Therapy of Idiopathic Pulmonary Fibrosis, EBioMedicine. (2025) 112, 10.1016/j.ebiom.2025.105538. PMC11754162 39753035

[21] Hawkins F. J. , Suzuki S. , Beermann M. L. et al., Derivation of Airway Basal Stem Cells From Human Pluripotent Stem Cells, Cell Stem Cell. (2021) 28, no. 1, 79–95.e78, 10.1016/j.stem.2020.09.017. 33098807 PMC7796997

[22] Zhou P. , Li Y. Q. , Zhang S. et al., KRT17 From Keratinocytes With High Glucose Stimulation Inhibit Dermal Fibroblasts Migration Through Integrin Alpha11, Journal of the Endocrine Society. (2024) 8, no. 2, 10.1210/jendso/bvad176. PMC10776312 38205163

[23] Li G. , Guo J. B. , Mou Y. F. et al., Keratin Gene Signature Expression Drives Epithelial-Mesenchymal Transition Through Enhanced TGF-β Signaling Pathway Activation and Correlates With Adverse Prognosis in Lung Adenocarcinoma, Heliyon. (2024) 10, no. 3, 10.1016/j.heliyon.2024.e24549. PMC10844058 38322947

[24] Kosykh A. V. , Ryumina I. I. , Botkina A. S. et al., EBS in Children With De Novo Pathogenic Variants Disturbing Krt14, International Journal of Molecular Sciences. (2024) 25, no. 5, 10.3390/ijms25052xxx. PMC10931735 38474236

[25] Ali F. M. , Zhou J. Y. , Wang M. Y. et al., Two Rare Case Reports of COL7A1 and EBS-GEN SEV KRT14 Variants With Review of Literature, BMC Pediatrics. (2024) 24, no. 1, 10.1186/s12887-024-04678-x. PMC10996244 38580989

[26] WalyEldeen A. A. , Sabet S. , Anis S. E. , Stein T. , and Ibrahim A. M. , FBLN2 is Associated With Basal Cell Markers Krt14 and ITGB1 in Mouse Mammary Epithelial Cells and has a Preferential Expression in Molecular Subtypes of Human Breast Cancer, Breast Cancer Research and Treatment. (2024) 208, no. 3, 673–686, 10.1007/s10549-024-07456-x. 39110274 PMC11522194

[27] Dong S. Q. , Wang L. L. , Liu X. Y. et al., CELF2 Inhibits Bladder Cancer Progression by Decreasing the Stability of CXCL5, Life Sciences. (2025) 370, 10.1016/j.lfs.2025.123585. 40154776

[28] Zhuo G. F. , Chen W. , Hu Y. N. et al., Genetic Prediction of the Phosphate-to-Glucose Ratio Mediates the Association Between CXCL5 and Vascular Dementia, Brain and Behavior. (2025) 15, no. 3, 10.1002/brb3.70378. PMC11938108 40135623

[29] Tong Y. , Chen Z. , Wu J. et al., METTL3 Promotes an Immunosuppressive Microenvironment in Bladder Cancer via m6A-Dependent CXCL5/CCL5 Regulation, Journal for Immunotherapy of Cancer. (2025) 13, no. 4, 10.1136/jitc-2024-xxxxx. PMC12001370 40234090

[30] Zhang H. and Yang B. , ADAM12 Silencing Mediated by FOXC2 Represses Meningioma Progression Through Inactivating the JAK1/STAT3/VEGFA Pathway, Biochemical Genetics. (2024) 10.1007/s10528-024-xxxxx-x. 39066954

[31] Mygind K. J. , Nikodemus D. , Gnosa S. et al., ADAM12-Generated Basigin Ectodomain Binds Beta1 Integrin and Enhances the Expression of Cancer-Related Extracellular Matrix Proteins, International Journal of Molecular Sciences. (2024) 25, no. 11, 10.3390/ijms25112xxx. PMC11172339 38892056

